# Differential cardiotoxic electrocardiographic response to doxorubicin treatment in conscious versus anesthetized mice

**DOI:** 10.14814/phy2.14987

**Published:** 2021-08-02

**Authors:** Anna Warhol, Sharon A. George, Sofian N. Obaid, Tatiana Efimova, Igor R. Efimov

**Affiliations:** ^1^ Department of Biomedical Engineering The George Washington University Washington DC USA; ^2^ Department of Anatomy and Cell Biology The George Washington University School of Medicine and Health Sciences Washington DC USA; ^3^ The GW Cancer Center The George Washington University School of Medicine and Health Sciences Washington DC USA

**Keywords:** anesthesia, doxorubicin, electrocardiography, isoflurane, sex differences

## Abstract

**Introduction:**

Doxorubicin (DOX), an anticancer drug used in chemotherapy, causes significant cardiotoxicity. This study aimed to investigate the effects of DOX on mouse cardiac electrophysiology, in conscious versus anesthetized state.

**Methods:**

Male and female C57BL/6 mice were injected with saline, 20 or 30 mg/kg DOX. ECGs were recorded 5 days post‐injection in conscious and isoflurane anesthetized states. ECGs were analyzed using a custom MATLAB software to determine P, PR, QRS, QTc, and RR intervals as well as heart rate variability (HRV).

**Results:**

ECGs from the same mouse demonstrated P wave and QTc shortening as well as PR and RR interval prolongation in anesthetized versus conscious saline‐treated mice. ECG response to DOX was also modulated by anesthesia. DOX treatment induced significant ECG modulation in female mice alone. While DOX20 treatment caused decrease in P and QRS durations, DOX30 treatment‐induced QTc and RR interval prolongation in anesthetized but not in conscious female mice. These data suggest significant sex differences and anesthesia‐induced differences in ECG response to DOX. HRV measured in time and frequency domains, a metric of arrhythmia susceptibility, was increased in DOX20‐treated mice compared to saline.

**Conclusions:**

This study for the first time identifies that the ECG response to DOX is modulated by anesthesia. Furthermore, this response demonstrated stark sex differences. These findings could have significant implications in clinical diagnosis of DOX cardiotoxicity.

## Graphical abstract

Doxorubicin treatment in mice produces acute cardiotoxic ECG response. Female sex and isoflurane anesthesia are associated with greater cardiotoxicity.

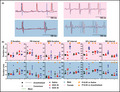

**NEW AND NOTEWORTHY**

This study for the first time identifies that sex and isoflurane anesthesia modulate the acute electrocardiographic response to doxorubicin treatment in mice. Isoflurane anesthesia unmasks acute ECG response to DOX, most notably in female mice. These findings can have significant impact on clinical diagnosis of acute cardiotoxicity in patients undergoing DOX chemotherapy.

## 1 INTRODUCTION

Doxorubicin (DOX) is an anthracycline antibiotic that is used in anticancer chemotherapy to treat several types of cancers including breast cancer, leukemia, and lymphoma (Trachtenberg et al., 2011). While DOX is very efficient in anticancer therapy, it has well‐established cardiotoxic effects and causes an increase in the development of fatal cardiotoxicity and heart failure (Chatterjee et al., 2010; Šimůnek et al., 2009; Swain et al., 2003).

DOX destroys cancer cells by inducing DNA fragmentation but it also has several detrimental side effects such as increased ROS production, inflammation, and antioxidant depletion which triggers the cellular stress signaling response (George et al., 2020; Ichikawa et al., 2014; Kim et al., 2006; Šimůnek et al., 2009). Several groups have identified the mechanistic pathways that are activated in this stress response to DOX and p38 MAPK activation and its role in promoting DOX cardiotoxicity is well established (Bernstein et al., 2005; George et al., 2020; Ma et al., 2012; Nozaki et al., 2004; Riad et al., 2008; Thandavarayan et al., 2010; Yi et al., 2007; Zhang et al., 2012). Activation of stress signaling pathways inhibits autophagy, increases apoptosis, fibrosis, cardiac dysfunction, and increases morbidity and mortality. Our group recently reported sex differences in the stress response to DOX treatment. Specifically, p38δ activation is associated with DOX‐induced cardiotoxicity in female mice (George et al., 2020).

DOX also affects cardiac electrophysiology and DOX‐induced changes in ion currents and action potentials have been previously reported (Fernandez‐Chas et al., 2018; Milberg et al., 2007; Sag et al., 2011; Wang et al., 2001; Wang & Korth, 1995). DOX decreases the delayed rectifier potassium current (I_Kr_) and increases the L‐type calcium current (I_CaL_) which results in action potential duration (APD) prolongation chronically, although DOX has been reported to acutely shorten APD (Fernandez‐Chas et al., 2018; Milberg et al., 2007; Sag et al., 2011; Wang et al., 2001; Wang & Korth, 1995). DOX also alters calcium handling and increases leak from the sarcoplasmic reticulum (Sag et al., 2011).

Due to numerous detrimental effects of DOX on cardiac function, it is crucial to identify techniques for early diagnosis of cardiotoxicity. Currently, echocardiography is the method of choice in the clinical setting and ejection fraction is the preferred metric (Meijers & Moslehi, 2019; Perez et al., 2019). However, in our previous study, we demonstrated the role of preserved ejection fraction in some subjects as a potential cause of undiagnosed DOX‐induced cardiotoxicity (George et al., 2020). Electrocardiography (ECG) is another method to detect DOX cardiotoxicity in patients, however, this technique too has its limitations. For example, the majority of ECGs recorded in a clinical setting are taken when the patient is in a conscious state. In contrast, most ECG recordings in animal models that were used to study and identify DOX cardiotoxicity were obtained from anesthetized mice. Anesthesia has also been shown to alter ECG parameters and decrease heart rate compared to conscious mice (Lindsey et al., 2018). Differences in physiological responses between conscious and anesthetized subjects and lack of robust correlation between studies in animal models to clinical testing, could complicate the diagnosis of acute cardiotoxicity in chemotherapy patients. This relationship, therefore, warrants a thorough investigation and is the objective of this study.

In this study, we determined the effects of DOX on cardiac electrophysiology and how it can be modulated by anesthesia. We report that DOX‐induced changes in ECG are more prominent in the anesthetized versus conscious state. Furthermore, we identified that sex is a significant variable that further alters this response.

## 2 METHODS

All protocols were approved by the Institutional Animal Care and Use Committee of the George Washington University and were in accordance with the National Institutes of Health Guide for the Care and Use of Laboratory Animals.

### 2.1 Mice

**FIGURE 1:**
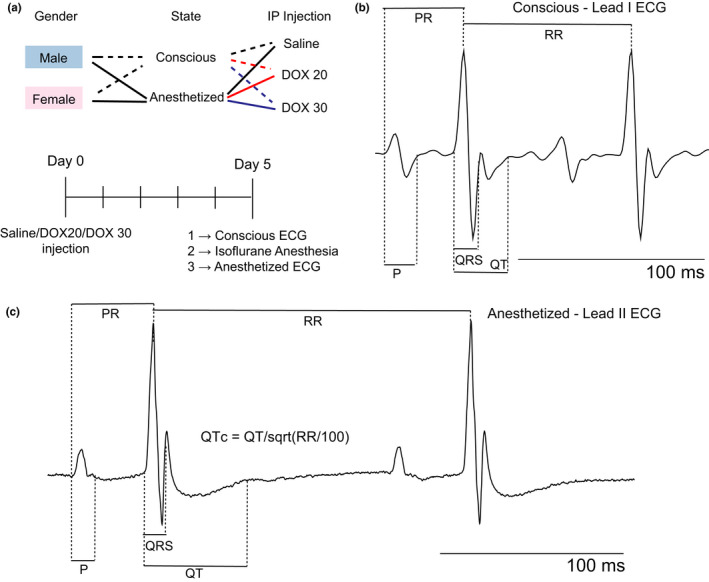
Methodology. (a) Different combinations of sex, anesthesia/conscious state, and saline/DOX injections used in this study are illustrated. These legends are consistent throughout the manuscript. Solid lines represent data from conscious mice and dashed lines from anesthetized mice. Data from saline‐treated mice are indicated in black, DOX20 in red, and DOX30 in dark blue. Pink highlighting represents data from female mice and blue from male mice. The timeline of the study including injections and data collection is illustrated below. (b) Representative ECG trace from a conscious (lead I) male saline‐treated mouse showing P, PR, QRS, QT, and RR intervals/durations measurements. (c) Representative ECG trace from an anesthetized (lead II) male saline‐treated mouse showing P, PR, QRS, QT, and RR intervals/durations measurements

Male and female mice on a C57BL/6 background, were intraperitoneally injected at ~15 weeks of age with either 20 mg/kg DOX (DOX20), 30 mg/kg DOX (DOX30), or saline (vehicle control) as shown in Figure 1A. Five days post‐injection, ECGs were recorded from the mice in the conscious and anesthetized state.

In conscious mice, ECG was recorded at 1 kHz sampling rate using the emka Technologies ecgTUNNEL device. Mice were guided into a restraining tunnel and positioned in prone position in contact with four silver ECG electrodes. After a 5‐minute acclimation period, conscious ECG recordings, approximately 2 min in duration, were recorded in lead I configuration (Figure 1B). Beyond 2 min, mice became stressed and motion artifacts were introduced into the signals. Mice were then anesthetized using isoflurane (1–2.5%, 1 ml/min oxygen) using an EZ anesthesia machine to maintain a heart rate of ~400 bpm (for echocardiography simultaneously performed and previously published (George et al., 2020)). Once unconscious, mice were transferred to the mouse platform of a Vevo 3100 ultrasound machine (FUJIFILM VisualSonics) and anesthesia administration was continued via a nose cone. A heated platform and heating lamp were used to maintain the body temperature of the mice during anesthesia. The limbs of the mice in supine position were taped to the four silver/silver chloride ECG electrodes on the platform and anesthetized ECG was recorded at a sampling rate of 8 kHz in lead II configuration (Figure 1C). Each file was of about 8 s in duration. ECG files were recorded along with the echocardiography files (data previously published (George et al., 2020)). Both conscious and anesthetized ECG traces were analyzed using the same custom MATLAB program and in the same manner as described below.

### 2.2 Electrocardiography Analysis

Conscious and anesthetized ECG data were exported to.txt and.csv file formats, respectively, and ECG parameters were measured using a custom MATLAB program (George et al., 2020). P, PR, QRS, QT, and RR intervals/durations were measured as illustrated in Figure 1B,C and as previously described (Boukens et al., 2014). QTc interval was calculated as $QTc\;=\;QT/\sqrt{RR/100}$.

### 2.3 Heart rate variability (HRV) analysis

Time and frequency domain HRV analysis were performed using a custom MATLAB program. Frequency domain HRV analysis was only performed on conscious mice because the anesthetized ECG recording was not of sufficient duration for this analysis.

#### 2.3.1 Time domain HRV analysis

A standard Poincaré plot was generated using consecutive RR intervals, RR_n_ versus RR_n+1_. SD1 and SD2 were measured as the short half axis and long half axis of an ellipse fitted to the Poincaré plot (Karmakar et al., 2009). SD1 represents short‐term variability and was calculated as ${SD1}^{2}\;=\;\frac{1}{2}{\mathrm{SDSD}}^{2}$, where SDSD is the standard deviation of the successive difference of RR interval. SD2 represents the long‐term variability and is calculated as ${SD2}^{2}\;=\;2{\mathrm{SDRR}}^{2}‐\frac{1}{2}{\mathrm{SDSD}}^{2}$, where SDRR is the standard deviation of RR interval (Karmakar et al., 2009; Shaffer & Ginsberg, 2017). SD1/SD2 ratio represents the autonomic balance.

#### 2.3.2 Frequency domain HRV analysis

Power spectral density (PSD) plot was created by plotting PSD against frequency. These measurements were once again calculated from the RR intervals. Power in the low‐ and high‐frequency (LF and HF, respectively) bands were calculated by taking the integral of the PSD in the LF and HF ranges previously reported for mouse HRV calculation (Gehrmann et al., 2000; Thireau et al., 2007). LF represents sympathetic activity and the frequency range used for LF was 0.1–1.5 Hz. HF represents the parasympathetic activity and frequency range used to measure HF was 1.5–4 Hz, as previously described (Thireau et al., 2007; Zila et al., 2015). The ratio of LF/HF represents an index of parasympathetic–sympathetic (autonomic) balance (Hsu et al., 2012). LF/HF correlates with the SD1/SD2 ratio in time domain analysis (Hsu et al., 2012). Similarly, SD1 correlates with HF and SD2 is closely correlated with LF.

### 2.4 Statistics

Statistical analyses were performed using GraphPad Prism software. All data are reported as mean ± standard deviation. Paired (conscious vs. anesthetized) and unpaired (Saline vs. DOX20/DOX30, males vs. females) Student's *t* tests were performed to determine statistical significance in differences between means and Bonferroni correction was applied to account for multiple comparisons. *P* < 0.05 denoted significance. Sample sizes for each data set are listed in the table and figure legends.

## 3 RESULTS

### 3.1 Isoflurane anesthesia modulates ECG

**FIGURE 2:**
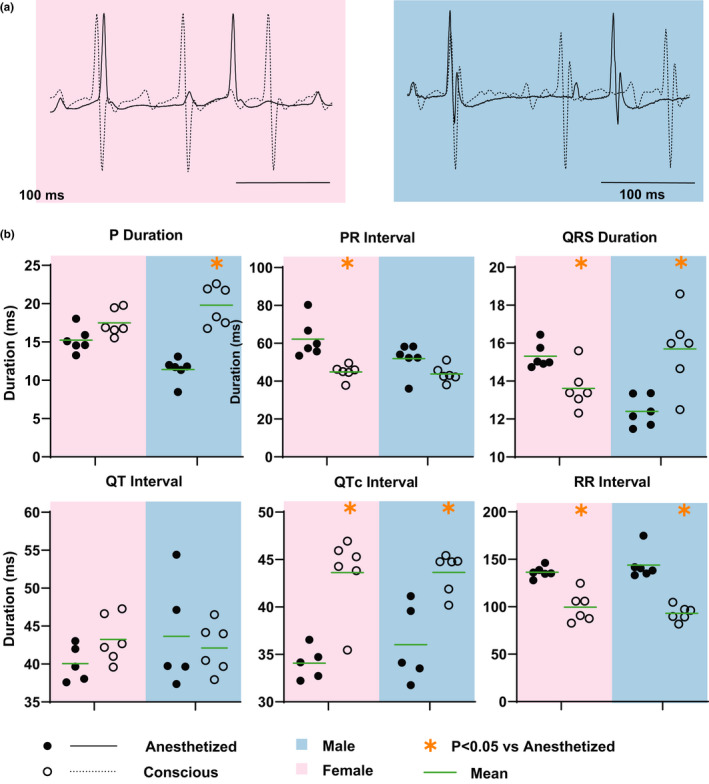
Isoflurane Anesthesia Modulates ECG. (a) Representative ECG traces from conscious (dashed line) and anesthetized (solid line) mice, male (blue) and female (pink), treated with saline. (b) Summary data of P wave duration, PR interval, QRS duration, QT interval, QTc interval, and RR interval measured from ECG of saline‐treated conscious (open circle) and anesthetized (filled circle), male and female mice. The mean for each group is represented by the green line. Statistics: Paired Student's *t* test with Bonferroni correction was performed to determine statistical significance between groups. *indicates *p* < 0.05 versus anesthetized mice. Sample size: *n* = 6 male and female mice

The effect of isoflurane anesthesia on ECG parameters in male and female mice was assessed with respect to conscious state of the same mice (Figure 2; Table 1). P wave duration, indicative of atrial excitation time, was shortened in male anesthetized versus conscious mice. On the other hand, PR interval, which denotes the atrioventricular (AV) delay, was prolonged in female anesthetized versus conscious mice but not in males. Interestingly, QRS duration, which indicates the ventricular excitation time, had opposite response in males and females. QRS duration was prolonged in female anesthetized versus conscious mice but shortened in male anesthetized versus conscious mice. QT interval was similar between the two states in males and females although RR interval prolongation in anesthetized mice resulted in shortened QTc intervals.

**TABLE 1:** Summary of data

Anesthetized mice						
	Female			Male		
	Saline	DOX 20	DOX 30	Saline	DOX 20	DOX 30
P (ms)	15.2 ± 1.5	11.2 ± 1.2**	11.6 ± 2.8	11.4 ± 1.4	11.6 ± 0.8	15.5 ± 7.2
PR (ms)	62.2 ± 9.1	58.7 ± 4.8	57.0 ± 7.0	51.9 ± 7.5	50.5 ± 4.1	63.7 ± 20.0
QRS (ms)	15.3 ± 0.6	13.1 ± 0.9 **	17.5 ± 4.9	12.4 ± 0.7	12.8 ± 2.5	14.4 ± 3.2
QT (ms)	40.0 ± 2.1	38.6 ± 12.6	82.8 ± 11.2 **	43.7 ± 6.3	54.5 ± 11.4	73.3 ± 35.9
QTc (ms)	34.1 ± 1.5	33.4 ± 10.9	60.3 ± 5.9 **	36.0 ± 3.7	46.1 ± 8.4	46.5 ± 15.6
RR (ms)	136.5 ± 5.4	138.2 ± 11.8	187.2 ± 24.3 **	144.0 ± 14.1	136.3 ± 16.2	218.5 ± 77.1
SD1 (ms)	0.8 ± 0.5	1.3 ± 1.1	2.1 ± 3.8	0.7 ± 0.3	4.8 ± 10.4	4.9 ± 4.9
SD2 (ms)	1.0 ± 0.7	1.3 ± 0.7	2.7 ± 5.9	0.9 ± 0.5	4.7 ± 9.8	5.4 ± 4.9
SD1/SD2	0.8 ± 0.1	0.9 ± 0.3	1.2 ± 0.8	0.9 ± 0.4	1.0 ± 0.3	0.9 ± 0.3

Conscious mice						
	Female			Male		
	Saline	DOX 20	DOX 30	Saline	DOX 20	DOX 30
P (ms)	17.5 ± 1.6	18.5 ± 3.8*	15.7 ± 3.0	19.8 ± 2.3 *	15.8 ± 4.5	18.2 ± 3.2
PR (ms)	44.9 ± 3.6 *	41.1 ± 3.6 *	42.8 ± 4.6 *	43.8 ± 4.0	39.7 ± 3.2 *	49.9 ± 11.5
QRS (ms)	13.6 ± 1.0 *	14.0 ± 1.0	16.6 ± 1.9 **	15.7 ± 1.8 *	13.6 ± 1.1	17.6 ± 4.2 *
QT (ms)	43.2 ± 2.8	45.4 ± 3.0	50.7 ± 1.4 * ^,^ **	42.1 ± 3.0	45.8 ± 3.5	52.4 ± 9.4
QTc (ms)	43.6 ± 3.8 *	45.5 ± 3.8 *	46.4 ± 3.4	43.6 ± 1.9 *	46.2 ± 2.6	46.3 ± 4.0
RR (ms)	99.6 ± 14.3 *	101.1 ± 14.8 *	122.2 ± 18.2 *	93.2 ± 7.3 *	99.8 ± 20.4 *	134.0 ± 50.2 *
SD1 (ms)	14.3 ± 6.5	25.6 ± 8.1 *	5.7 ± 4.9	8.9 ± 5.0	15.2 ± 7.6	13.7 ± 8.2
SD2 (ms)	17.9 ± 8.6	38.9 ± 14.5 * ^,^ **	6.7 ± 5.7	15.9 ± 9.6	19.9 ± 10.9	18.8 ± 11.0
SD1/SD2	0.8 ± 0.1	0.7 ± 0.1 *	0.9 ± 0.3	0.6 ± 0.2	0.8 ± 0.3	0.7 ± 0.2
LF (ms^2^)	1.8 ± 1.3	7.8 ± 6.0	1.0 ± 2.2	1.2 ± 1.3	3.0 ± 2.6	12.7 ± 23.7
HF (ms^2^)	2.8 ± 2.2	5.0 ± 4.1	2.1 ± 4.8	0.8 ± 1.0	2.5 ± 1.9	10.3 ± 20.1
LF/HF	0.7 ± 0.2	1.7 ± 0.9	0.3 ± 0.2 **	2.2 ± 1.4	2.2 ± 2.6	1.5 ± 0.8

The mean ± standard deviation of all ECG and HRV parameters from each experimental group is listed. The data sets are separated by anesthetized versus conscious, sex, and then by treatment (saline, DOX20, or DOX30).

Abbreviations: HF, High‐frequency HRV; LF, Low‐frequency HRV; LF/HF, Ratio of low‐ and high‐frequency HRV; P, P wave duration; PR, PR interval; QRS, QRS duration; QT, QT interval; QTc, QTc interval; RR, RR Interval; SD1, Standard deviation of short‐term HRV; SD1/SD2, Ratio of short‐ and long‐term HRV; SD2, Standard deviation of long‐term HRV.

Sample sizes for each group are as follows: Females––Conscious Saline: n = 6, Conscious DOX20: n = 10, Conscious DOX30: n = 7, Anesthetized Saline: n = 6, Anesthetized DOX20: n = 10, Anesthetized DOX30: n = 10 and Males––Conscious Saline: n = 6, Conscious DOX20: n = 8, Conscious DOX30: n = 8, Anesthetized Saline: n = 6, Anesthetized DOX20: n = 8, Anesthetized DOX30: n = 8.

* indicates *p* < 0.05 versus anesthetized mice (paired).

** indicates *p* < 0.05 versus saline‐treated group (unpaired).

### 3.2 DOX‐induced ECG changes are modulated by anesthesia

**FIGURE 3:**
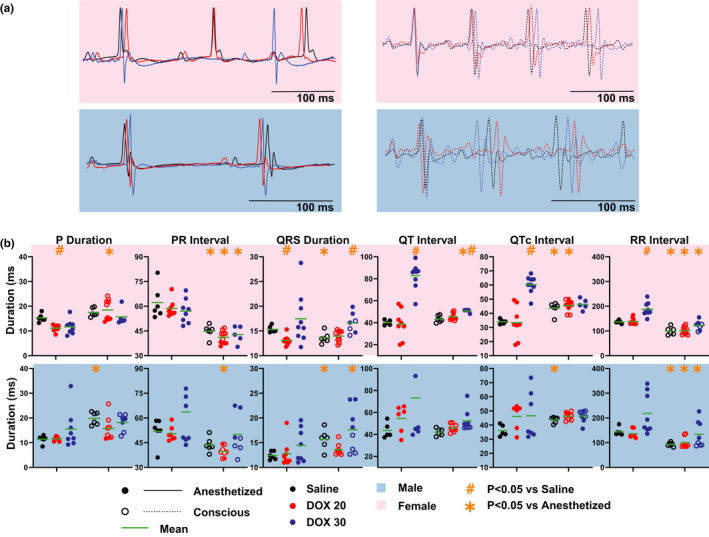
ECG response to DOX treatment is modulated by Isoflurane Anesthesia. (a) Representative ECG traces of anesthetized and conscious mice treated with saline (black), DOX20 (red), and DOX30 (dark blue) are shown. (b) Summary data of P wave duration, PR interval, QRS duration, QT interval, QTc interval, and RR interval measured from ECG of conscious (open circle) and anesthetized (filled circle), male and female mice. The mean for each group is represented by the green line. Statistics: Paired and unpaired Student's *t* test with Bonferroni correction were performed to determine statistical significance between groups. *indicates *p* < 0.05 versus anesthetized mice (paired) and #indicates *p* < 0.05 versus saline‐treated group (unpaired). Sample sizes for each group are as follows: Females––Conscious Saline: *n* = 6, Conscious DOX20: *n* = 10, Conscious DOX30: *n* = 7, Anesthetized Saline: *n* = 6, Anesthetized DOX20: *n* = 10, Anesthetized DOX30: *n* = 10 and Males––Conscious Saline: *n* = 6, Conscious DOX20: *n* = 8, Conscious DOX30: *n* = 8, Anesthetized Saline: *n* = 6, Anesthetized DOX20: *n* = 8, Anesthetized DOX30: *n* = 8

ECG response in DOX‐treated anesthetized and conscious mice was compared next and is illustrated/summarized in Figure 3 and Table 1. DOX and isoflurane anesthesia, individually and in combination, induced more changes in female ECGs compared to males. First, looking at the effects of DOX alone in conscious mice, DOX30 induced QRS and QT interval prolongation in female mice. No changes in ECG parameters were measured in male mice or those treated with DOX20. Next, in anesthetized mice, with the combination of DOX and isoflurane, DOX20 shortened P and QRS durations while DOX30 treatment prolonged QT, QTc, and RR intervals in females. Once again, DOX did not alter any ECG parameters in anesthetized males.

Lastly, we compared ECG parameters between conscious and anesthetized mice within the same treatment group (DOX20 and DOX30). In DOX20‐treated mice, isoflurane anesthesia caused P wave and QTc interval shortening, and PR and RR interval prolongation among females, while in males PR and RR interval prolongation was observed. Similar effects were observed with DOX30 treatment. Additionally, anesthesia prolonged the QT interval in female DOX30‐treated mice, and QRS duration in males.

To summarize, DOX induced the most significant changes in ECG parameters in female anesthetized mice compared to all other groups studied here. This indicates that there are differences in ECG response between the conscious and anesthetized states, males and females, during DOX treatment.

### 3.3 Sex differences in ECG response to DOX

**FIGURE 4:**
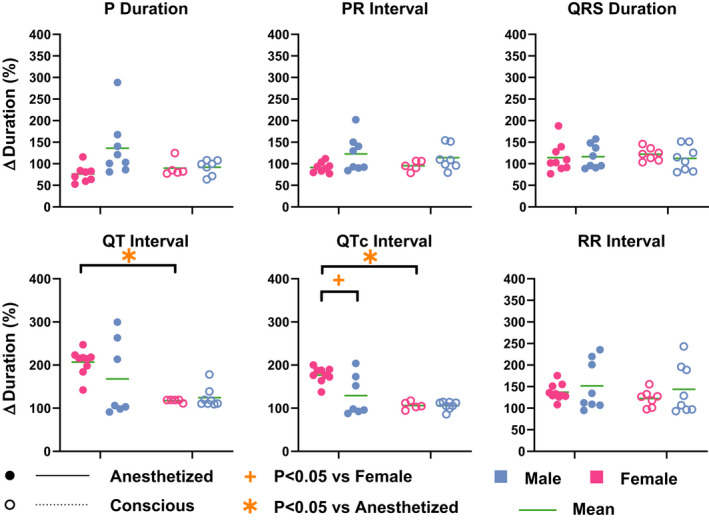
Sex differences in ECG response to DOX treatment. Percent change in ECG parameters in DOX30‐treated mice relative to saline‐treated mice is illustrated here. Changes in P wave duration, PR interval, QRS duration, QTc interval, and RR interval are reported for female (pink) and male (blue) mice conscious (open circle) and anesthetized (filled circles) states. Green line represents the mean of the data. Statistics: Paired and unpaired Student's *t* test with Bonferroni correction were performed to determine statistical significance between groups. *indicates *p* < 0.05 versus anesthetized mice (paired) and +indicates *p* < 0.05 versus female mice (unpaired). Sample sizes for each group are as follows: Females––Conscious: *n* = 7, Anesthetized: *n* = 10, and Males––Conscious: *n* = 8, Anesthetized: *n* = 8

Sex differences in response to DOX were investigated next. ECG parameters in DOX30‐treated mice were normalized to its own saline control (Figure 4). First, when comparing between the anesthetized and conscious mice within the same sex, QT and QTc intervals were significantly prolonged in anesthetized versus conscious females, while no significant differences were measured in males.

Sex differences in ECG response to DOX were also observed. QTc interval was significantly prolonged in anesthetized females compared to males. A similar trend is also observed in QT interval, however, the presence of three very sick mice in the anesthetized male group appears to limit the statistical significance of this metric. Taken together, these results further demonstrate that DOX‐induced ECG modulation is more pronounced in female mice than in males.

### 3.4 Time domain HRV analysis

**FIGURE 5:**
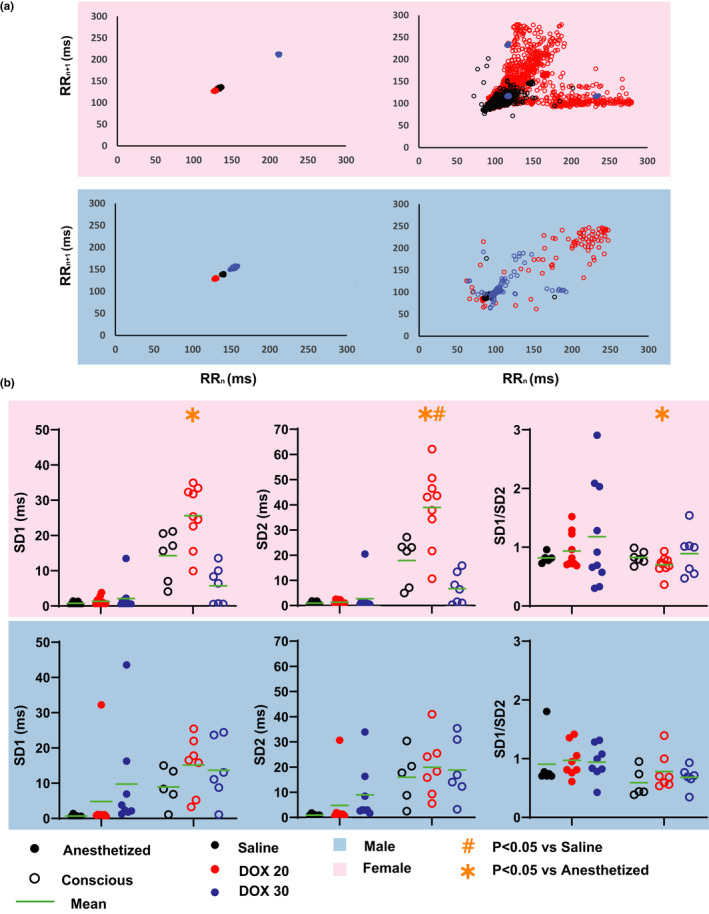
Time Domain HRV Analysis. (a) Poincaré plots for anesthetized (left, filled circles) and conscious (right, open circles) mice show heartrate variability in saline (black)‐, DOX20 (red)‐, and DOX30 (blue)‐treated mice. (b) SD1, SD2, and SD1/SD2 ratios are reported for each group. Statistics: Paired and unpaired Student's *t* test with Bonferroni correction were performed to determine statistical significance between groups. *indicates *p* < 0.05 versus anesthetized mice (paired) and #indicates *p* < 0.05 versus saline‐treated group (unpaired). Sample sizes for each group are as follows: Females––Conscious Saline: *n* = 6, Conscious DOX20: *n* = 10, Conscious DOX30: *n* = 10, Anesthetized Saline: *n* = 6, Anesthetized DOX20: *n* = 9, Anesthetized DOX30: *n* = 7 and Males––Conscious Saline: *n* = 6, Conscious DOX20: *n* = 8, Conscious DOX30: *n* = 8, Anesthetized Saline: *n* = 6, Anesthetized DOX20: *n* = 8, Anesthetized DOX30: *n* = 6

HRV was next measured from Poincaré plots as illustrated in Figure 5. There is little dispersion in beat‐to‐beat RR intervals in anesthetized mice, which indicates very little HRV even after DOX treatment (Figure 5A, Left). On the other hand, there is more dispersion in the conscious Poincaré plots (Figure 5A, Right), especially when the mice were treated with DOX. SD1 and SD2 were significantly increased in conscious versus anesthetized female mice treated with DOX20. Additionally, comparing DOX to saline‐treated mice, DOX20 treatment further increased SD2 in conscious female mice which in turn decreased the SD1/SD2 ratio. No significant changes in the time domain HRV parameters were measured in males.

To summarize, while DOX‐induced changes in the ECG parameters discussed above are more prominent in the anesthetized mice, DOX‐induced changes in HRV are more prominent in conscious mice. However, in both instances, female mice displayed more pronounced cardiotoxic ECG response compared to males.

### 3.5 Frequency domain HRV analysis

**FIGURE 6:**
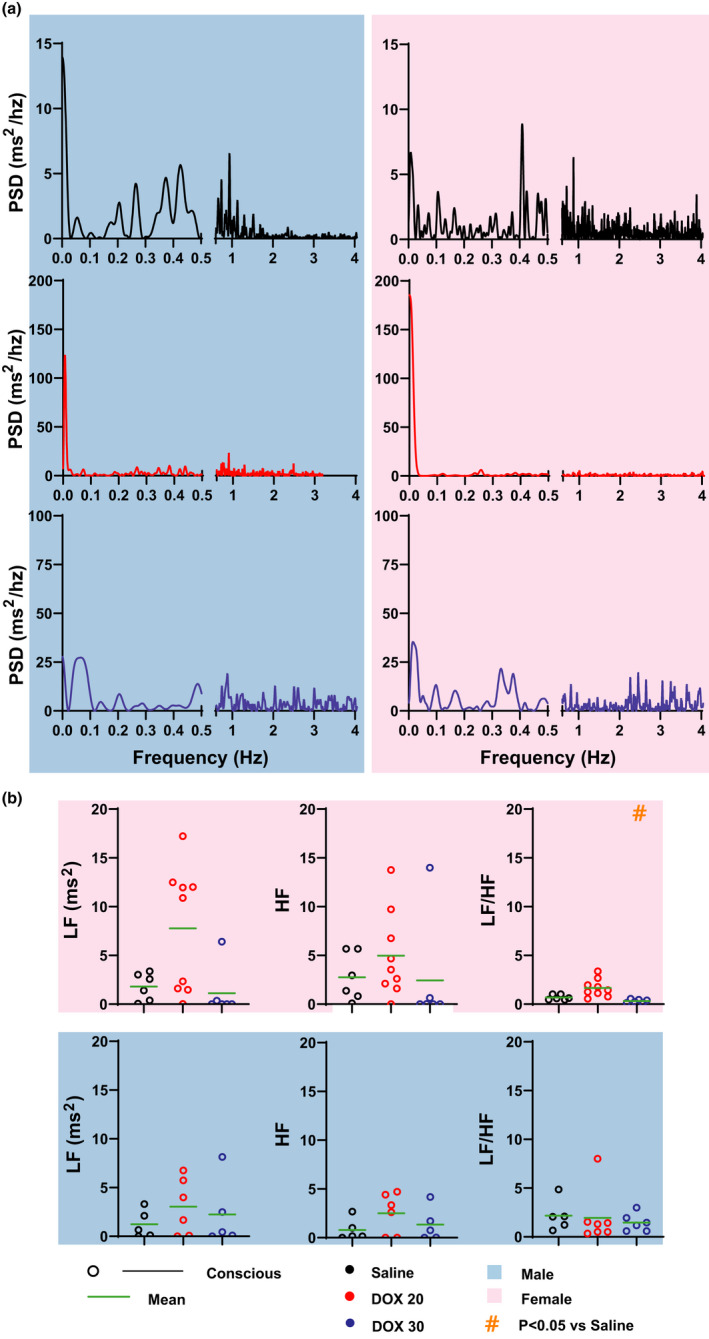
Frequency Domain HRV Analysis. (a) Power spectra of heart rate variability in conscious mice treated with saline (black), DOX20 (red), and DOX30 (blue). (b) Power in low‐ (LF, 0.1–1.5 Hz) and high‐frequency (HR, 1.5–4 Hz) bands and its ratio are reported for conscious saline‐, DOX20‐, and DOX30‐treated mice. Statistics: Unpaired Student's *t* test with Bonferroni correction was performed to determine statistical significance between groups. #indicates *p* < 0.05 versus saline‐treated group. Sample sizes for each group are as follows: Females––Conscious Saline: *n* = 6, Conscious DOX20: *n* = 10, Conscious DOX30: *n* = 10 and Males––Conscious Saline: *n* = 6, Conscious DOX20: *n* = 8, Conscious DOX30: *n* = 8

Frequency domain HRV analysis was performed only on conscious mice ECG due to the limited duration of anesthetized mice ECG. DOX20 treatment in female mice increased LF and HF compared to saline (Figure 6), although it was not significant due to the larger variability in these parameters. Of note, two clusters of LF values were observed in DOX20‐treated females suggesting two different responses to DOX treatment. On the other hand, DOX30 treatment did not alter LF or HF parameters in males or females even though LF/HF ratio was significantly reduced in females. This reduction was due to a non‐significant reduction in LF, suggesting reduced sympathetic tone in these mice.

## 4 DISCUSSION

In this study, the role of isoflurane anesthesia in modulating ECG response to DOX treatment was investigated in male and female mice. We report here that isoflurane by itself modulates ECG parameters and furthermore, also alters ECG response to DOX treatment in a sex‐dependent manner. ECG modulation in response to DOX treatment was most significant in female anesthetized mice. Time and frequency domain HRV analyses indicated increased HRV in conscious compared to anesthetized mice, with DOX treatment further increasing HRV. These findings uncover a significant variable that can confound the translation of basic cardiotoxicity research, primarily performed in anesthetized laboratory animals, to clinical acute and chronic cardiotoxicity studies performed in conscious patients. Additionally, it highlights the importance of taking into consideration sex differences in determining cardiotoxic ECG response in laboratory and clinical settings.

### 4.1 Effects of isoflurane on ECG:

Isoflurane is a commonly used anesthetic in mice because it is stable, has a short induction time, and has no long‐term effects on mice (Lindsey et al., 2018; Ludders, 1992). Isoflurane anesthesia has several direct and indirect (via modulating autonomic tone) effects on cardiac physiology. It is well established that isoflurane slows heart rate and, therefore, prolongs RR interval in ECGs (Constantinides et al., 2011; Lindsey et al., 2018). Consistent with previous studies, we observed prolonged RR intervals in male and female anesthetized mice relative to conscious mice. We additionally also report here that isoflurane anesthesia modulated P wave duration, PR interval, QRS duration, and QTc interval.

P wave duration is indicative of the time taken for atrial excitation, and PR interval denotes the time taken for electrical excitation to propagate from the atria to the ventricles via the AV node. P wave duration shortening in male mice observed in this study suggests faster electrical excitation propagation in the atria of anesthetized mice. On the other hand, prolonged PR interval in anesthetized females could suggest increased AV delay in these mice during isoflurane anesthesia.

QRS duration represents the ventricular excitation time. Interestingly, we observed opposite effects of isoflurane anesthesia on QRS duration in male and female mice. While isoflurane prolonged QRS duration in female mice, it shortened QRS duration in male mice. Factors that can contribute to such acute changes in ventricular excitation could include ion channel function modulation, particularly the depolarizing sodium current. Isoflurane has been reported to inhibit the fast sodium current which could result in QRS prolongation as observed in the female mice in this study (Weigt et al., 1997). However, sex differences in the effects of isoflurane on I_Na_ have not been reported and the basis for QRS shortening in male mice will require further investigation.

Isoflurane alters multiple cardiac ion currents and can also alter APD (Hüneke et al., 2004). Isoflurane inhibits T‐ and L‐type calcium currents which can shorten APD (Hüneke et al., ,,^2001^, ^2004^; Orestes et al., 2009). On the other hand, isoflurane also inhibits the transient outward potassium current and delayed rectifier potassium current which can prolong APD (Hüneke et al., 2001; Suzuki et al., 2003). The opposing effect of these ion currents inhibition on APD could be the source of the mixed results in literature on the effects of isoflurane on APD. While some studies report APD shortening, others report a dose‐dependent biphasic APD response to isoflurane exposure. Macroscopic APD modulation typically manifests as changes in QT interval. In this study, we report no change in QT interval even though significant QTc shortening was observed in isoflurane anesthetized mice due to isoflurane‐induced bradycardia. The insensitivity of the mouse QT interval to heart rate was recently reported (Mulla et al., 2018; Roussel et al., 2016; Speerschneider & Thomsen, 2013) which calls into question the rate correction of the QT interval. In this study, we report both QT and QTc intervals because while we did not see rate‐dependent changes in QT interval in healthier mice (Figure 2); in sicker DOX30‐treated mice with severe bradycardia (Figure 3), we recorded a similarly prolonged QT and QTc intervals.

Thus, the combined effect of isoflurane on cardiac ion channels functioning could contribute to the ECG modulation as described in this study. It is, however, unknown at this point if these effects of isoflurane anesthesia are a direct result of isoflurane altering cardiac electrophysiology or an indirect effect due to altering the autonomic tone. Future studies will distinguish these mechanisms.

### 4.2 Effects of DOX on ECG:

DOX has numerous detrimental effects on cardiac function. The acute and chronic effects of DOX on the ECG have been investigated previously. We previously reported significant cardiac dysfunction and reduced survival rate (40% in males and 90% in females) in DOX30‐treated mice (George et al., 2020). ECGs from most DOX‐treated animal models have previously been performed under anesthesia, which, as we have shown here, can additionally alter cardiac function. Therefore, we first discuss our results in anesthetized mice to compare with previous studies.

A previous study reports that DOX causes an increase in QT interval and ST durations as well as prolongation of the QRS complex (significant and non‐significant) in anesthetized male Wistar rats (Hazari et al., 2009). Chronic DOX administration (a dose of 3.75 mg/kg at week 2, 3, 4, and 5 to make a cumulative dose of 15 mg/kg) has also been reported to decrease heart rate (Hazari et al., 2009). Consistent with these findings, we report here that DOX30 treatment prolonged QT and QTc intervals and reduced heart rate (increased RR interval) in anesthetized mice, however, these effects were only observed in female mice and not in males. In contrast to previous studies, we report in this study that DOX treatment resulted in significantly shortened QRS duration in female anesthetized mice with no change in males. This discrepancy could be due to species‐specific differences (rats vs. mice) or due to the additional effect of the anesthetic used (mild ether anesthetic vs. isoflurane). Finally, we also observed P duration shortening after DOX treatment, once again, only in female anesthetized mice. Thus, to summarize, in anesthetized female mice, acute effects of DOX include faster conduction of electrical impulses in the atria and ventricles (short P and QRS), prolonged repolarization (QT), and slowed heart rate.

Previous studies that performed conscious ECG recordings in rats, report DOX‐induced prolongation of PR interval, QRS duration, QTc interval, and ST interval (Hazari et al., 2009; Jensen et al., 1984). Consistent with these studies, we also observed QRS and QT prolongation in female conscious mice. Interestingly, the effect of DOX on QRS duration in female mice was opposing in conscious versus anesthetized states in the present study. While QRS duration was shortened by DOX in female anesthetized mice, it was prolonged in conscious state. This is the first study to directly compare the effects of DOX on male and female ECGs. These sex‐ and anesthesia‐induced differences could have a significant impact on acute cardiotoxicity diagnosis in the clinical setting.

Finally, a dose‐dependent HRV response was observed in DOX‐treated mice where DOX20 increased HRV parameters, whereas DOX30 had similar HRV parameters compared to saline‐treated mice. This biphasic response could be a result of reduced variability but more drastically modulated ECG parameters in the much sicker DOX30‐treated mice.

### 4.3 Sex differences in ECG response to DOX

Previous human studies have demonstrated that there are sex differences in the recording of electrical activities in the heart (Meiners et al., 2018; Mieszczanska et al., 2008; Ravens, 2018). These studies show prolonged PR interval and QRS duration and shortened QT interval in males compared to females. These studies also indicated slower heart rate in males compared to females.

Our present study for the first time identifies sex differences in ECG of anesthetized and conscious mice. While anesthesia in DOX30‐treated female mice unmasked multiple ECG parameter modulations, no changes were observed in males. Interestingly, we demonstrated in our previous study (George et al., 2020) that cardiac dysfunction was more prevalent in DOX30‐treated males compared to females. Thus, examining DOX treatment in mice uncovered sexual dimorphism in the ensuing cardiotoxicity and showed that cardiac electrical and mechanical functions were differently affected in males versus females.

Previous clinical studies have also identified that male sex is associated with increased risk of cardiotoxicity in adults (Meiners et al., 2018; Myrehaug et al., 2008, 2010). Mouse studies have also reported increased mortality among males treated with DOX compared to females (George et al., 2020; Meiners et al., 2018; Moulin et al., 2015). In contrast to these findings, we report here the acute modulation of more ECG parameters in female mice compared to males treated with DOX20 and DOX30. This sexual dimorphism in ECG response to DOX is a significant new finding that will impact the diagnosis of cardiotoxicity in patients. Separate measures of acute ECG responses to DOX treatment will need to be considered for the diagnosis of cardiotoxicity in patients.

### 4.4 Clinical Implications

The findings of this study indicate that the state of consciousness of the subject can alter diagnostic parameters of DOX cardiotoxicity. More specifically, anesthesia unmasked acute ECG cardiotoxic response in DOX‐treated female mice. Note that this does not warrant anesthetizing patients undergoing chemotherapy, and we caution against it. However, these findings have several important clinical implications. First, they suggest that a lack of cardiotoxic ECG response to DOX treatment in conscious patients may not correspond to a lack of cardiotoxicity. Cardiotoxic response may be more evident during a state of unconsciousness such as during sleep. Therefore, continuous monitoring of patients undergoing chemotherapy may be more suitable to identify acute cardiotoxicity. Second, while DOX cardiotoxicity is more prevalent in adult males (mice and humans) versus females (George et al., 2020; Meiners et al., 2018; Myrehaug et al., 2008, 2010), the findings presented here suggest that this may not be associated with significant ECG changes acutely. On the other hand, significant ECG changes were observed in female mice which exhibited lesser cardiac dysfunction and greater survival. This suggests that cardiotoxicity takes different forms between the sexes and diagnostic approaches must take these sex differences into account. Finally, these findings also highlight the lack of robust correlation between basic science research models and clinical data and underscore the need to identify and utilize models that more comprehensively mimic clinical conditions (in this case, anesthetized vs. conscious state).

### 4.5 Limitations

This study employs a single dose, intraperitoneal injection of saline, DOX20, or DOX30, 5 days prior to the conscious and anesthetized ECG recording. While this is a well‐established model for studying DOX cardiotoxicity in mice it is not representative of clinical chemotherapy regimens (George et al., 2020). Future studies will implement multiple smaller doses of DOX injections to study chronic cardiotoxicity manifestation in the ECG.

While we recorded ECGs in lead II configuration from anesthetized mice, lead I configuration was implemented for conscious mice due to differences in the devices used to record ECGs. This difference in ECG configurations can affect the morphology and some measurements of ECG parameters measured here. A previous study reported ~7 ms difference in QT intervals of patients measured from the two ECG leads (Salvi et al., 2012). However, this relatively small lead‐based difference is unlikely to change the larger anesthesia‐ and DOX‐induced ECG modulation reported in this study. Nonetheless, it is important to identify the difference in other ECG parameter measurements.

The study also recorded shorter ECG traces in the anesthetized mice compared to the recordings of conscious mice due to differences in devices used. As a result, it was not possible to perform frequency domain HRV analysis on the anesthetized ECGs. There was not enough data to accurately estimate the low‐frequency ranges and power spectra from an anesthetized mouse ECG recording which was about 8 s in length.

Finally, isoflurane by itself can modulate ECG as shown in Figure 1. Isoflurane can exert these effects on cardiac electrophysiology directly or indirectly by altering physiologic functions such as the autonomic balance. While this study simply attributes the observed ECG modulation to the state of anesthesia, future studies aimed at distinguishing between the mechanisms underlying isoflurane anesthesia, particularly in the presence of DOX, are crucial.

## 5 CONCLUSIONS

We demonstrated here the independent and interdependent effects of (1) isoflurane anesthesia, (2) DOX treatment, and (3) sex in modulating mouse cardiac electrophysiology. While the heart rate slowing effect of isoflurane anesthesia is well‐known, we have performed a thorough characterization of the mouse ECG response to isoflurane. We additionally identified that anesthesia can unmask the effects of DOX treatment on the ECG. This could have important implications in the diagnosis and treatment of DOX‐induced cardiotoxic effects in the clinical setting. This finding is all the more significant because ECG collected from mice and other animal models in laboratory settings are mostly collected under anesthesia, while ECG acquisition in the clinical setting is performed in conscious patients. This could lead to masking of the ECG alterations to DOX treatment in these conscious patients, and highlight a lack of synchrony in basic science versus clinical research. Therefore, it is crucial to take into account conscious versus anesthetized state of the subjects in ECG studies.

## AUTHOR CONTRIBUTIONS

SAG, TE, and IRE conceptualized and designed the study, SAG and SNO performed the experiments, AW, SAG, and SNO analyzed the data, AW and SAG prepared the manuscript, all authors edited and approved the manuscript.
